# Small Molecule Soluble Epoxide Hydrolase Inhibitors in Multitarget and Combination Therapies for Inflammation and Cancer

**DOI:** 10.3390/molecules25235488

**Published:** 2020-11-24

**Authors:** Amarjyoti Das Mahapatra, Rinku Choubey, Bhaskar Datta

**Affiliations:** 1 Department of Chemistry, Indian Institute of Technology Gandhinagar, Palaj, Gandhinagar 382355, India; amarjyoti.mahapatra@iitgn.ac.in (A.D.M.); rinku.choubey@iitgn.ac.in (R.C.); 2 Department of Biological Engineering, Indian Institute of Technology Gandhinagar, Palaj, Gandhinagar 382355, India

**Keywords:** soluble epoxide hydrolase (sEH) inhibitors, urea derivatives, inflammation, combination chemotherapy, multitarget therapy

## Abstract

The enzyme soluble epoxide hydrolase (sEH) plays a central role in metabolism of bioactive lipid signaling molecules. The substrate-specific hydrolase activity of sEH converts epoxyeicosatrienoic acids (EETs) to less bioactive dihydroxyeicosatrienoic acids. EETs exhibit anti-inflammatory, analgesic, antihypertensive, cardio-protective and organ-protective properties. Accordingly, sEH inhibition is a promising therapeutic strategy for addressing a variety of diseases. In this review, we describe small molecule architectures that have been commonly deployed as sEH inhibitors with respect to angiogenesis, inflammation and cancer. We juxtapose commonly used synthetic scaffolds and natural products within the paradigm of a multitarget approach for addressing inflammation and inflammation induced carcinogenesis. Structural insights from the inhibitor complexes and novel strategies for development of sEH-based multitarget inhibitors are also presented. While sEH inhibition is likely to suppress inflammation-induced carcinogenesis, it can also lead to enhanced angiogenesis via increased EET concentrations. In this regard, sEH inhibitors in combination chemotherapy are described. Urea and amide-based architectures feature prominently across multitarget inhibition and combination chemotherapy applications of sEH inhibitors.

## 1. Arachidonic Acid Pathway and Epoxyeicosatrienoic Acids (EETs)

Arachidonic acid (ARA) is an omega-6 polyunsaturated fatty acid (PUFAs) and metabolized by three major classes of enzymes including cyclooxygenases (COXs), lipoxygenases (LOXs, and cytochrome P450s (CYPs), resulting in the formation of various inflammatory mediators such as prostaglandins, prostacyclin, lipoxins and leukotrienes [1]. Through the cytochrome P450s pathways, ARA can be converted to two kinds of eicosanoid acids: epoxyeicosatrienoic acids (EETs) by cytochrome P450 (CYP) epoxygenase, and hydroxyeicosatetraenoic acids (HETEs) by CYP α-oxidases [2]. The cytochrome P450-derived HETEs are proinflammatory mediators, whereas EET is vasodilatory towards specific cells and tissues. Soluble epoxide hydrolase (sEH) is a prominent enzyme involved in the degradation of anti-inflammatory EETs and is considered as the major pathway inactivating EETs to corresponding diols inducing the infiltration of proinflammatory mediators. Expression of both pro and anti-inflammatory mediators are influenced by various factors such as rheumatic diseases, myocardial infarction, angina, aging, obesity and pharmacotherapy [3]. Enhanced inflammatory cell infiltration causes chronic inflammation and inflammation-induced carcinogenesis. Thus, sEH targeting is commonly suggested for preventing inflammation-induced carcinogenesis [4].

The CYP2C and CYP2J enzymes convert arachidonic acid to four EET regioisomers, namely 5,6-EET, 8,9-EET, 11,12-EET, and 14,15-EET [5,6]. Elevated EET levels in breast cancer tissues are associated with upregulation of specific CYPs, and downregulation of sEH, and are also associated with aggressive cell behavior in patients [7]. EETs are also reported to promote the pathogenesis of various human cancers [8,9,10].

## 2. Soluble Epoxide Hydrolase (sEH)

Epoxide hydrolases have been detected in prokaryotes and eukaryotes ranging from plants to mammals [11,12,13]. In mammals, these include soluble epoxide hydrolase (sEH), microsomal epoxide hydrolase (mEH), cholesterol epoxide hydrolase, and leukotriene A4 (LTA4) hydrolase. These enzymes mediate the addition of water to both exogenous and endogenous epoxides, leading to the corresponding vicinal diols [14]. sEH effectively utilizes 8,9-, 11,12-, and 14,15-EETs, while 5,6-EET is a poor substrate.

sEH is composed of two structurally and functionally independent domains with two distinct activities. The C-terminal domain is responsible for hydrolase activity, while the N-terminal domain shows phosphatase activity [15]. While sEH exhibits broad tissue distribution, high levels of sEH are found in intestine, liver, kidney, brain and vasculature with lower levels in lung, spleen and testes [5]. In human hepatocytes and renal proximal tubules, sEH localizes to both the cytosol and peroxisomes. However, it is exclusively cytosolic in other sEH-containing cells such as pancreatic islet cells, intestinal epithelium, anterior pituitary cells, adrenal gland, endometrium, lymphoid follicles, prostate ductal epithelium and blood vessels [5].

The C-terminus epoxide hydrolase motif of sEH transforms four regioisomers of EETs, namely, 5,6-, 8,9-, 11,12-, and 14,15-EETs, to the corresponding dihydroxyeicosatrienoic acids (DHETs), whereby the biological effects of EETs are diminished, eliminated or altered [16]. Spectroscopic studies and crystallographic studies have revealed the catalytic site and reaction mechanism of epoxide ring opening activity by sEH [17,18]. The reaction takes place via an SN2-type reaction based on the cooperative action of a catalytic triad composed of Tyr382, Tyr465, and Asp334. Tyr382 and/or Tyr465 form hydrogen bonds with the epoxide oxygen and activate the epoxide of the substrate through polarization. Nucleophilic attack by Asp334 of the enzyme on an epoxide carbon, usually at the least sterically hindered and most reactive carbon, leads to the formation of a covalent hydroxyl-alkyl enzyme intermediate. The nucleophile Asp334 is oriented and activated by a His523-Asp495 pair. Subsequently, a water molecule activated by His523 attacks the alkyl-enzyme intermediate to form a tetrahedral intermediate, which finally collapses to release the corresponding diol product [19,20]. Mutation of either tyrosine to a phenylalanine leads to a 90% loss of enzyme activity [19]. A recent study based on molecular dynamics simulation suggests that the protonation of His523 is essential for the proper orientation of Asp333 [21]. sEH hydrolase activity has been shown to be dependent on sEH dimerization. Disrupting sEH dimerization may serve as a novel therapeutic strategy for reducing sEH hydrolase activity [22].

## 3. Role of EETs in Angiogenesis and Inflammation

Organisms are routinely exposed to endogenous and exogenous epoxide-containing compounds such as EETs. An epoxide (or oxirane) possesses distinctive reactivity patterns owing to the highly polarized oxygen-carbon bonds in addition to a highly strained ring [20]. Reactive epoxides such as 8,9-, 11,12-, 14,15-EETs, and some exogenous epoxides, are responsible for electrophilic reactions with DNA and proteins, leading to mutagenic, toxic and carcinogenic effects [20].

EETs generate an anti-inflammatory effect on the endothelium by inhibiting cytokine-induced NF-κB transcription factor [23]. The transcription factor NF-κB is a key component of the cellular response to damage, stress and inflammation. NF-κB activation is mediated by inhibitory kappa B (IκB) kinase (IKK), which is, in turn, activated in response to a variety of pathogenic factors. Activated IKK phosphorylates an inhibitory protein, IκB, which results in IκB degradation and thereby allows NF-κB to translocate to the nucleus and to induce the synthesis of proinflammatory molecules [24]. EETs block the inhibitor of nuclear factor-κB (IκB) kinase IKK-mediated phosphorylation of IkBα, maintaining NF-κβ in an inactive state [21]. Considering that EETs are secreted from endothelial cells, a natural connect is expected between EETs and angiogenesis [25,26,27,28,29]. The signaling pathway that forms this connect varies depending on the species, type of endothelium and the EET regioisomer responsible for initiating the process [24]. Overall, three signaling pathways play a role in EET-mediated angiogenesis [24]. The first pathway is a cAMP-dependent pathway that activates the cAMP response-element binding protein (CREBP) and COX-2 expression. This pathway is activated by EETs produced by CYP2C9, especially 11,12- EET [30]. The second pathway is also activated by EETs produced by CYP2C9, but involves PI3K and Akt, leading to an increase in cyclin D1 expression [26]. The third pathway is a p38 MAPK pathway that is activated by 8,9-EET and 11,12-EET [31].

**Figure 1:**
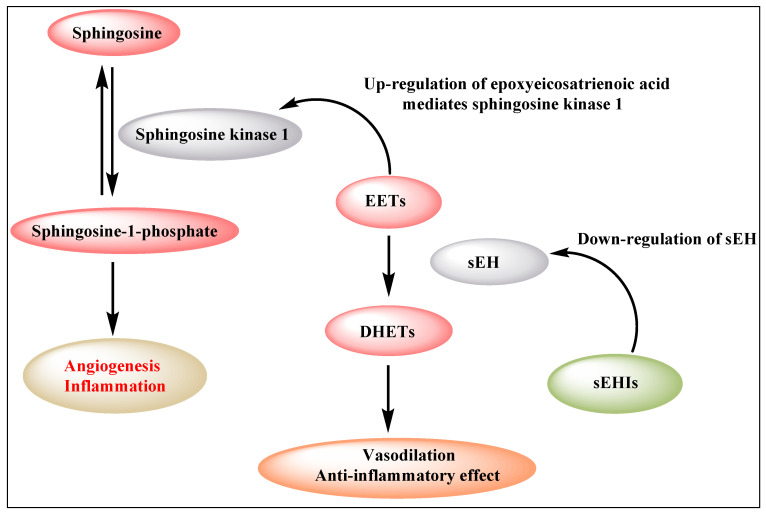
Biphasic effect of EETs (EETs: epoxyeicosatrienoic acids; sEH: soluble epoxide hydrolase; DHETs: dihydroxyeicosatrienoic acids).

The sphingosine kinases (SphK1 and SphK2) are a distinctive group of lipid kinases responsible for ATP-dependent phosphorylation of sphingosine to produce Sphingosine-1-phosphate (S1P). SphK1 has emerged as a significant signaling enzyme because of its contribution to the growth, metastasis and chemoresistance of various human cancers [32,33]. Sphingosine kinase 1 (SphK1) participates in the activation of the inflammatory response via sphingosine-1-phosphate formation. Notably, SphK1 is also an important mediator of 11,12-EET induced angiogenic pathways in human endothelial cell (EC) [34]. The expression of a dominant-negative SphK1 or knockdown of SphK1 by siRNA, inhibited 11,12-EET-induced endothelial cell proliferation, migration, tube formation in vitro and Matrigel plug angiogenesis in vivo [34]. 14,15-EET is reported to induce angiogenesis through several pathways and receptors, including TRPV1 [35], Src, PI3K/Akt signaling in parallel with mTOR-S6K1 activation and Src-dependent STAT-3-mediated VEGF expression [24]. These observations point to a biphasic effect of EETs (Figure 1). In particular, inhibition of sEH, and the subsequent increase in EET concentration, results in enhanced angiogenesis, thereby stimulating primary tumor growth and metastasis.

## 4. sEH in Inflammation-Driven Carcinogenesis

**Figure 2:**
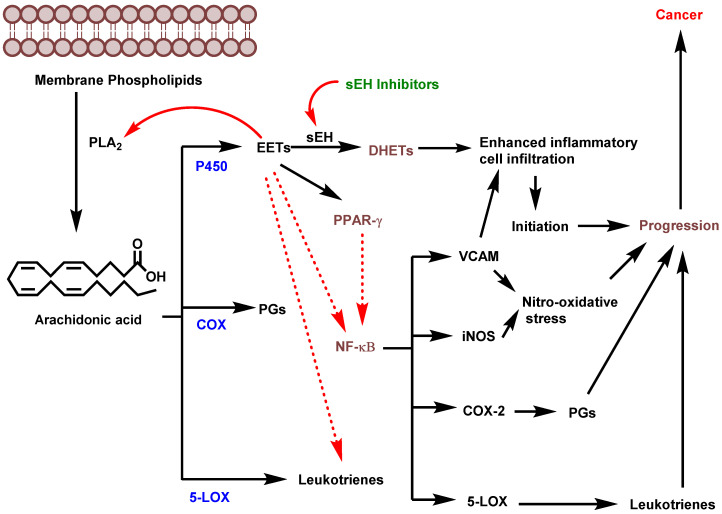
Potential role of sEH in inflammation-driven carcinogenesis (PLA2: phospholipase A2; EETs: epoxyeicosatrienoic acids; sEH: soluble epoxide hydrolase; DHETs: dihydroxyeicosatrienoic acids; COX: cyclooxygenase; LOX: lipoxygenase; PGs: prostaglandins; PPAR-γ: Peroxisome proliferator-activated receptor gamma; NF-κβ: (nuclear factor kappa-light-chain-enhancer of activated B cells; VCAM: vascular cell adhesion molecule; iNOS: Inducible nitric oxide synthase). (Figure adapted from [5]).

Materials inflammation-driven cancer is based on the infiltration of white neutrophilic granulocytes, and involvement of cytokines and chemokines [36]. Soluble epoxy hydrolase is a prominent proinflammatory enzyme that is responsible for the infiltration of inflammatory mediators. Inhibition of sEH significantly reduces the conversion of EETs to corresponding DHETs, thereby highlighting sEH as an important target for addressing inflammation and carcinogenesis [4]. Node et al. demonstrated that physiological concentrations of EETs or overexpression of CYP2J2 inhibist inflammation by decreasing cytokine-induced endothelial cell adhesion molecule expression, and preventing leukocyte adhesion to vascular walls and tissues by a mechanism involving inhibition of transcription factor NF-kB and IkB kinase [23]. Inhibition of inflammation is suggested based on multiple pathways that result in suppressing inflammatory cell recruitment, modulating the arachidonic acid metabolite profile and further targeting the PPAR-gamma and NF-kB pathways, which, in turn lead to an inhibition of COX-2, 5-LOX, iNOS and VCAM-1 (Figure 2) [5].

## 5. Soluble Epoxide Hydrolase Inhibitors (sEHIs)

Soluble epoxide hydrolase inhibitors (sEHIs) have been leveraged for addressing a variety of diseases including hypertension [37,38,39], cancer [7,25,40], dyslipidemia [41], pain [42,43,44,45,46], immunological disorders [47], neurological diseases [45,48,49,50], diabetes [51,52,53,54] and eye diseases [55,56]. Urea, carbamate and amide derivatives represent the most potent class of pharmacophores reported as sEHIs [57,58,59]. Crystal structures of urea derivatives bound to sEH revealed that the carbonyl oxygen of the urea group accepts hydrogen bonds from Tyr residues (381, 465), and the nitrogen atom of NH group donates a hydrogen bond to Asp333. Urea derivatives mimic the transition state for epoxide ring opening and exert their inhibitory property [60]. Based on the L-shaped hydrophobic pocket of sEH, lipophilic functional groups, such as adamantyl, biphenyl or halogens, can enhance the potency of urea-based inhibitors. Crystal structure of nonurea inhibitors, for example benzoxazole amide and fulvestrant, bound to sEH have been reported [61,62]. Xing and coworkers applied structure-based virtual screening to design combinatorial libraries to discover benzoxazole based novel and potent soluble epoxide hydrolase (sEH) inhibitors. The benzoxazole nitrogen atom and the amide oxygen atom form concurrent hydrogen bonds with Tyr381 and Tyr465, respectively. The benzoxazole ring nitrogen atom and the amide nitrogen form hydrogen bonds with His523 and Asp333, respectively [61]. In contrast, fulvestrant complexed with sEH showed that the oxygen atom of the sulfoxide (S=O) group forms hydrogen bonds with tyrosines 383 and 466 [62]. Interestingly, a hydrogen bond interaction between the sulfur atom of sulfoxide group and Asp335 was also observed. 

**Figure 3:**
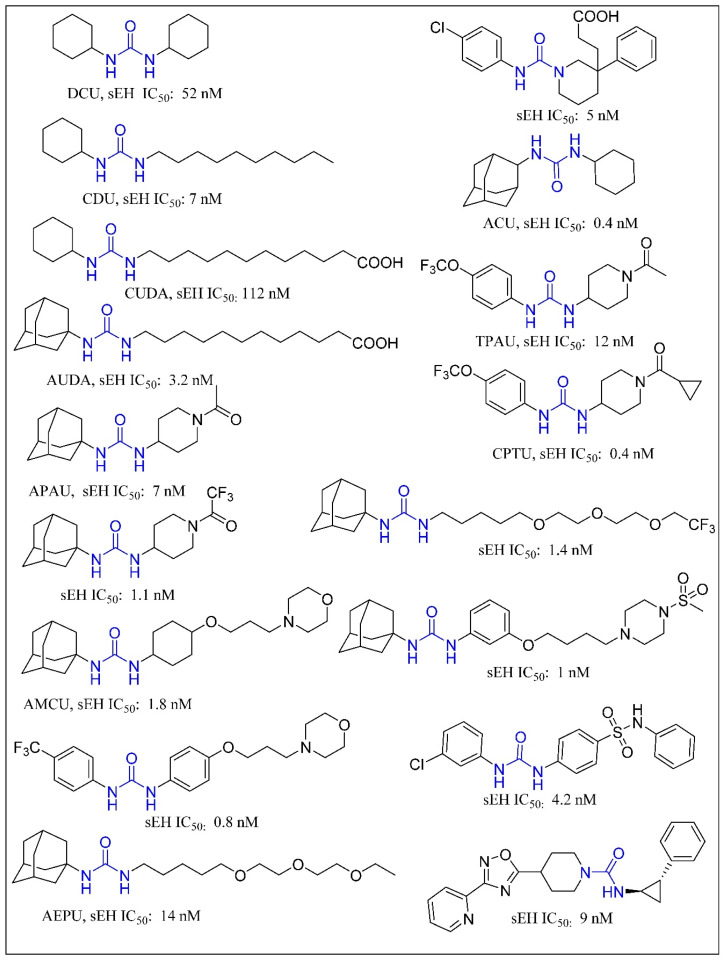
Urea based soluble epoxide hydrolase inhibitors.

**Figure 4:**
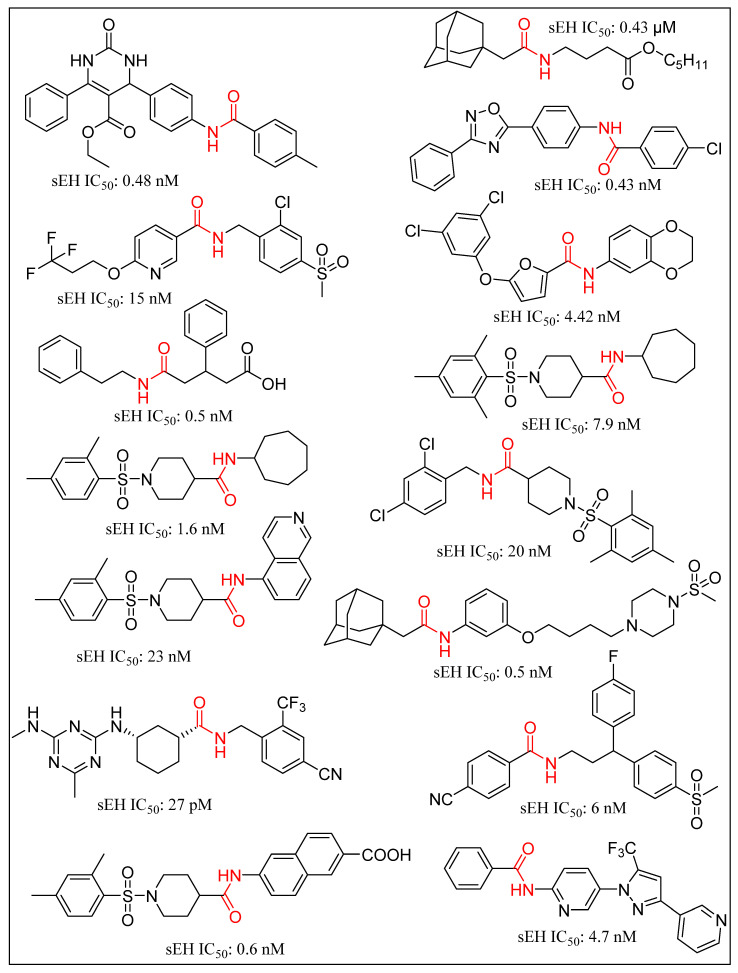
Amide based soluble epoxide hydrolase inhibitors.

Trans-3-phenylglycidols and chalcone oxides were first reported as sEHIs, and it was demonstrated that substituted chalcone oxides are more potent inhibitors of sEH enzyme than trans-3-phenylglycidols [63,64,65]. A large number of urea derivatives have been shown to possess sEH inhibitory activity (Figure 3) [66,67,68,69,70]. One of the earliest urea derivative DCUs showed excellent in vivo inhibitory activity against sEH enzyme, albeit constrained by unfavorable pharmacokinetic properties [16]. Similarly, another urea-based potent inhibitor CDU was hampered by rapid metabolization in hepatic microsomes [71]. Fatty acid analogs, AUDA displayed excellent sEH inhibitory activity but were restricted in their applicability due to fatty acid β-oxidation and P450 oxidation-based instability of the adamantane group. A higher accumulation of fatty acid epoxides, and a relatively lower amount of the corresponding diol, was observed in urine after 24 h of treatment of AUDA in AngII-infused rats [72]. Substitution of a long alkyl chain by a polar group positioned approximately 7 Å from the urea carbonyl group improved the physical properties of inhibitors leading to incorporation of the ether functional group in AEPU [73]. Replacement of ether chain by conformationally restricted substituents as in APAU, c-AUCB, t-AUCB, and TPPU resulted in favorable pharmacokinetic properties along with low nanomolar potency [38,74,75,76,77]. Several trisubstituted urea derivatives have been reported with excellent potency and attractive bioavailability [77,78,79,80]. Urea derivatives containing fluorene and adamantane groups increased the sEH inhibitory activity by a factor of 4.5 [81]. The replacement of adamantyl and 4-(trifluoromethoxy)phenyl groups with natural bicyclic lipophilic groups provided a series of sEHIs with similar potency and 10-fold more water solubility compared to the original compounds [82]. On a similar note, Codony and coworkers developed 2-oxaadamant-1-yl urea-based molecules and reported that the replacement of a methylene unit of the adamantane group by an oxygen atom increased the solubility, permeability, and stability with nanomolar sEH inhibitory potency [83]. Pharmacophore-based virtual screening has been used for discovery of 6-amino-2-((4-chlorobenzyl)thio)-5-phenylpyrimidin-4(3H)-one and urea-based potent sEHIs [84]. Although structure-activity relationship (SAR) studies provided a significant number of selective and potent urea-based sEHIs, their low solubility and poor metabolic stability continue to hamper pharmacological use in vivo. A variety of amide and α-keto or α-hydroxy amide derivatives are recognized as potent sEHIs (Figure 4) [59,85,86,87,88,89,90,91,92,93,94].

**Figure 5:**
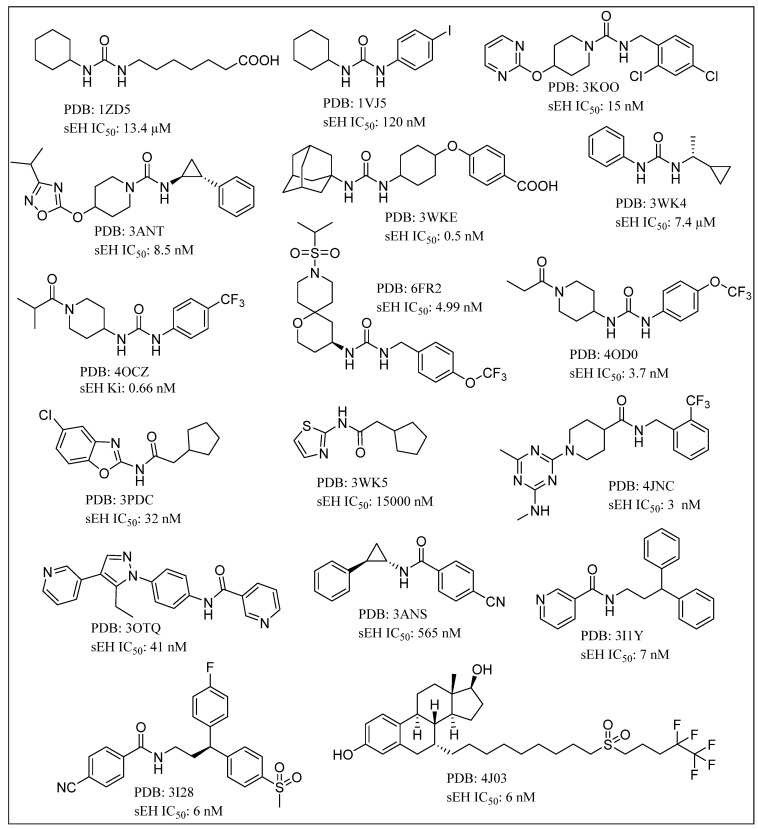
Chemical structure of small molecule inhibitors of human soluble epoxide hydrolase (sEH) in X-ray crystal structure complexes with PDB and IC_50_ values.

Other moieties that have shown promising sEHI activity include aminobenzisoxazole, acyl hydrazones, 4-benzamidobenzoic acid hydrazide, adamantyl thioureas and sulphoxide derivatives [61,62,95,96,97,98] The ligand-based pharmacophore model has recently led to identification of novel N-benzoyl-2-phenylhydrazine-1-carboxamide and ethyl (R)-6-methyl-2-(3-(p-tolyl)ureido)-4,5,6,7-tetrahydrobenzo[b]thiophene-3-carboxylate as potential sEHIs with IC_50_ values of less than 5 µM [99]. A variety of small molecule inhibitors of human sEH with reported X-ray crystal structures (PDB ID) and IC_50_ values are depicted in Figure 5 [15,58,59,61,62,78,100,101,102,103,104,105]. The chemical space for sEHIs has been further expanded by the recent work of Scholz and coworkers on demonstration of carboranes as non-natural 3-D pharmacophores and sEH inhibition by carboranylcarboxamide compounds [106].

**Figure 6:**
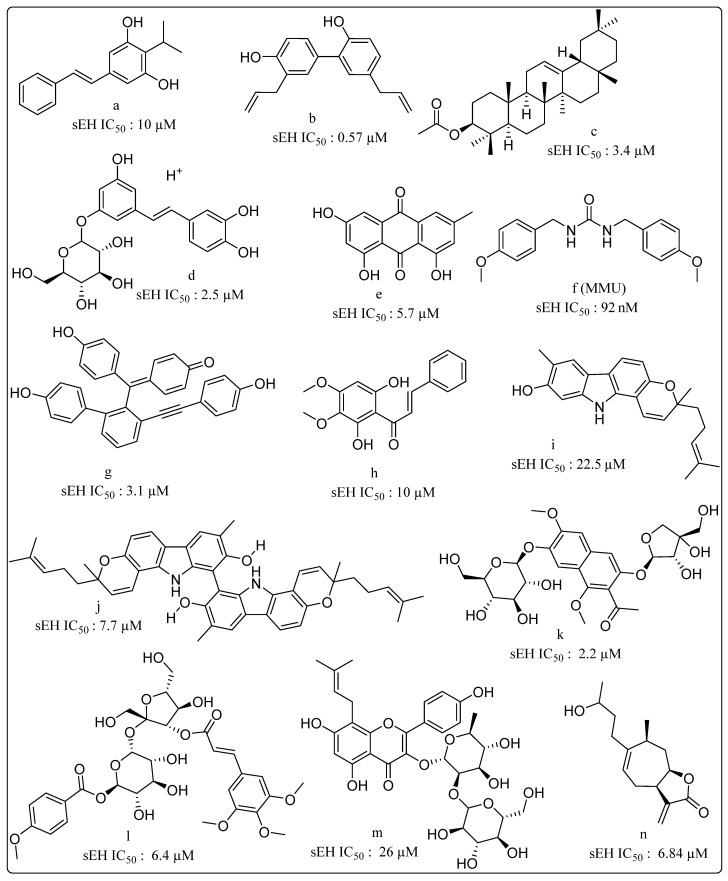
Natural products displaying soluble epoxide hydrolase (sEH) inhibitory properties (**a**) Isopropylstilbene from *Photorhabdus*, (**b**) Honokiol isolated from *Magnolia officinalis*, (**c**) β-amyrin acetate isolated from *Acer mandshuricum*, (**d**,**e**) from *Rheum undulatum*, (**f**) MMU from *Pentadiplandra brazzeana Baillon*, (**g**) Selaginellin derivatives from *Selaginella tamariscina*, (**h**) (E)-1-(2,6-dihydroxy-3,4-dimethoxyphenyl)-3-phenylprop-2-en-1-one from *Docynia indica* (*Wall*.) *Decne*. (**i**) Isomahanine and (**j**) Bisisomahanine from the aerial parts of *Glycosmis stenocarpa*, (**k**) the seeds of *Cassia tora*, (**l**) phenolic glycosides from *Polygala tenuifolia*. (**m**) Prenyl-flavonoids from *Epimedium koreanum* Nakai (**n**) 4H-tomentosin from *Inula helenium*.

Research on natural product-based sEHIs development has sought to address the typical drawbacks of synthetic small molecule inhibitors. A plethora of natural products, including stilbenes, carbazole, anthraquinones, selaginellin, cycloartane triterpene, capsaicin, dihydrocapsaicin, anthocyanin derivatives and phenolic glycosides derivatives, possess sEHI activity (Figure 6) [107,108,109,110,111,112,113,114,115,116,117,118,119]. Interestingly, very few natural products bear the urea or carbamate groups that form the mainstay of synthetic sEHIs. Moracin and coumarin derivatives from mulberry leaves have been identified as significant sEHIs [120]. Sun and coworkers collected protostane-type triterpenoids from *Alisma orientale* and reported their potential sEH inhibitory activities [121]. Natural products have also provided a platform for exploration of mimics towards sEH inhibition. A series of partially saturated analogues containing epoxide bioisosteres were synthesized and evaluated for in vitro inhibition of sEH by Falck and coworkers [122]. Similarly, a new generation of EET mimics incorporating modifications to the carboxylate were prepared and evaluated for inhibition of sEH [123]. A number of frontier approaches, such as DNA-encoded small-molecule libraries, have been adopted towards identification of sEHIs [124]. Belyanskaya and coworkers deployed the encoded library technology (ELT) for the discovery and early clinical development of an inhibitor of the enzyme soluble epoxide hydrolase (GSK2256294). This particular platform combines DNA encoding, combinatorial organic chemistry and affinity-based selection, and can efficiently yield high quality molecules with favorable pharmacological properties [125].

Giancarlo and coworkers developed a distinctive dual inhibitory mechanism of the endogenous 15-deoxy-Δ12,14 prostaglandin J2 (15d-PGJ2) whereby hsEH can be inhibited by reversible docking of 15d-PGJ2 in the catalytic pocket, as well as by covalent locking of the same compound onto cysteine residues C423 and C522 remote to the active site. This mode of inhibition expands the scope of development of new allosteric inhibitors of hsEH [126].

## 6. Dual Inhibition/Modulation of sEH as Part of Anti-Inflammatory Therapeutics

Soluble epoxide hydrolase inhibitors have been studied in combination with other target-specific inhibitors as potential multitarget therapies. The consideration of sEH in anti-inflammatory drug development is notable in this regard. Nonsteroidal anti-inflammatory drugs (NSAIDs) and selective cyclooxygenase 2 (COX-2) inhibitors (coxibs), which are capable to block COX-2 mediated conversion of ARA to prostaglandin E2 (PGE2), are widely used to treat inflammation and pain [127]. NSAIDs are associated with several gastrointestinal injury-based adverse effects including ulceration, bleeding, inflammation and even perforation [128].

**Figure 7:**
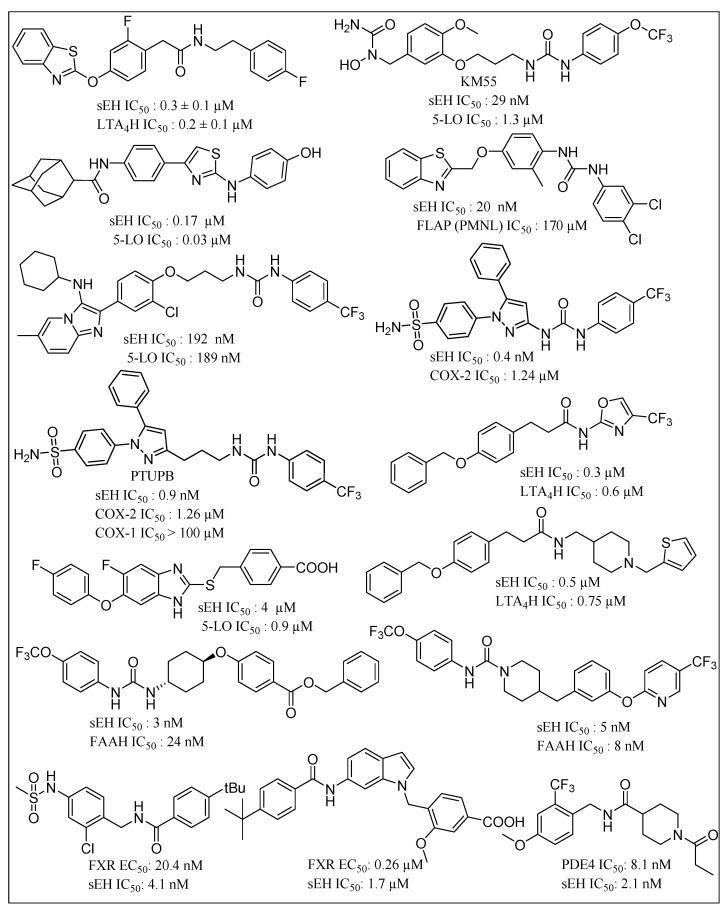
Chemical structures of small molecule based dual COX-2/sEH inhibitor, 5-LO/sEH inhibitor, FLAP/sEH inhibitor, sEH/ LTA4H inhibitors, sEH/FAAH inhibitors, sEH/PDE4 inhibitors, and FXR activator/sEH inhibitor. (COX: cyclooxygenase; sEH: soluble epoxide hydrolase; 5-LO: 5-lipoxygenase; FLAP: 5-lipoxygenase activating protein; LTA4H: leukotriene A4 hydrolase; FAAH: fatty acid amide hydrolase; PDE4: phosphodiesterase 4; FXR: Farnesoid X receptor).

sEHIs have been suggested as being capable of synergistic reduction of the side effects of NSAIDs [129,130,131]. Inhibition of EET catabolism via sEHIs, results in a significant increase in their anti-inflammatory properties, providing scope for effective treatment of inflammatory disease and associated cancers. Synergistic activity of sEHIs with COX inhibitors are effective against obesity-induced colonic inflammation. The latter is a major risk factor for colorectal cancer, inflammatory bowel disease, chronic colitis and pancreatitis. A variety of amide and urea-based small molecule dual sEHIs have been reported in this context (Figure 7). Hwang et al. reported urea-containing pyrazole derivatives as dual inhibitors of cyclooxygenase-2 and sEH [132]. Novel urea-diarylpyrazole hybrids exhibit dual COX-2/sEH inhibition with improved anti-inflammatory activity and highly reduced cardiovascular risks [133]. Leukotrienes (LTs) are proinflammatory lipid mediators, regulate the innate immune response and play a pathophysiological role in chronic inflammatory diseases such as asthma and atherosclerosis. The 5-lipoxygenase activating protein (FLAP) facilitates ARA conversion by 5-lipoxygenase (5-LO) to proinflammatory leukotrienes (LTs). Inhibition of FLAP has been reported to efficiently abolish LT formation in vitro and in vivo. Thus, dual inhibition of FLAP and sEH is likely to represent a powerful anti-inflammatory strategy due to simultaneous suppression of proinflammatory LTs and dihydroxyeicosatrienoic acids while maintaining anti-inflammatory EETs [134]. Garscha et al. have reported the in vivo pharmacological profile of difapolin that dually targets FLAP and sEH and are promising for treatment of inflammation-related diseases [135]. Pharmacophore-based virtual screening has been used for developing dual inhibitors of the 5-lipoxygenase-activating protein and sEH [136]. Notably, N-[4-(benzothiazol-2-ylmethoxy)-2-methylphenyl]-N′-(3,4-dichlorophenyl)urea shows an IC_50_ of 200 nM in a cell-based FLAP test system and 20 nM for sEH activity in a cell-free assay. It was demonstrated that LTA4H specifically converts the instable epoxide LTA4 toward the proinflammatory LTB4. Thus, dual inhibition of LTA4H and sEH is likely to have superior effects in resolving inflammation due to accumulation of the anti-inflammatory EETs and blockage of the chemoattractant LTB4. Hiesinger et al. developed amide derivatives with a benzothiazole core as a dual inhibitor of sEH and LTA4 hydrolase, thereby demonstrating another future route for development of anti-inflammatory agents [137]. Hefke et al. deployed computer-aided fragment growing strategies to design amide based dual inhibitors of sEH and LTA4 hydrolase [138]. Other strategies in this regard include benzimidazole derivatives as dual sEH/5-Lipoxygenase inhibitors using virtual screening [139]. Substituted fluorobenzimidazoles derivatives have been reported as dual inhibitors of 5-lipoxygenase and sEH [140]. A hybrid imidazo-[1,2a]-pyridine-based dual inhibitor of sEH and 5-lipoxygenase exhibits high potency in vitro [141]. Other sEH/5-LO dual inhibitors include fragment-based development of 2-aminothiazole derivatives [142]. Meirer et al. developed a dual 5-LO/sEH inhibitor KM55 which significantly inhibits the LPS-induced adhesion of leukocytes to endothelial cells by blocking leukocyte activation [143]. Dual inhibitors for sEH and fatty acid amide hydrolase (FAAH) have been shown to synergize responses to inflammatory and neuropathic pain [144,145] Dual inhibitors focused on sEH have sought to leverage the role of targets that are implicated in regulation of inflammation. For example, sEH and p38β kinase have been demonstrated as two key regulators of inflammation. TPPU has been reported as both a human sEH and p38β kinase inhibitor with nanomolar potencies and distinct selectivity [146]. On a similar theme, Farnesoid X receptor (FXR) activation is associated with antisteatotic and antifibrotic effects which promise synergies with a sEHI based anti-inflammatory strategy. Schmidt and coworkers developed an amide-based dual modulator of farnesoid X receptor and soluble epoxide hydrolase to counter nonalcoholic steatohepatitis [147]. Recently, dual FXR activators/sEH inhibitors have been reported that are derived from the antiasthma drug Zafirlukast. These inhibitors possess favorable dual potency towards FXR and sEH while reducing the original cysteinyl leukotriene receptor antagonism of the lead drug [148]. Inceoglu and coworkers demonstrated that coadministration of phosphodiesterase 4 (PDE4) and sEH inhibitors results in an enhanced analgesic effect compared to the individual treatments [149]. Further, bioavailable sEH/PDE4 dual inhibitors have been reported for management of pain as well as tools for investigating the biology of pain [150].

## 7. Dual Inhibition of sEH in Combination Chemotherapy

Chemotherapy promotes tumorigenesis, angiogenesis and metastasis via apoptotic tumor cell-induced macrophage chemotaxis and proinflammatory cytokines [151,152,153]. Gartung and coworkers reported that ovarian tumor cell debris generated by first-line platinum and taxane-based chemotherapy accelerates tumor progression by stimulating a macrophage-derived surge of proinflammatory cytokines and bioactive lipids [154]. Targeting the debris-mediated surge of protumorigenic factors, including inflammatory cytokines, chemokines, proangiogenic growth factors and danger signals (e.g., alarmins), offers an approach for enhancing the efficacy of cytotoxic therapies. The combined pharmacological abrogation of the cyclooxygenase-2 (COX-2) and sEH pathways has been shown to prevent the debris-induced surge of both cytokines and lipid mediators by macrophages. PTUPB prevents the chemotherapy-induced cytokine/lipid surge and suppresses debris-stimulated ovarian tumor growth by acting as a surge protector against therapy-induced protumorigenic mediators, ultimately improving patient survival by preventing tumor recurrence [154].

**Figure 8:**
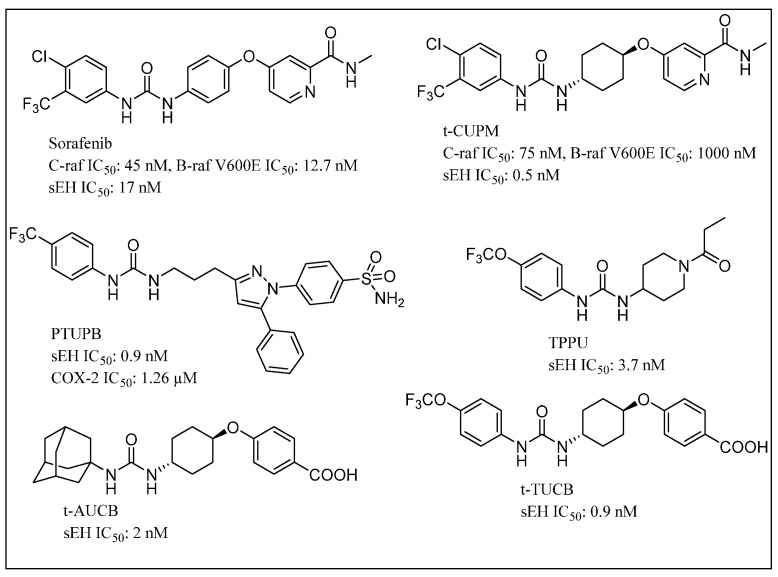
Chemical structures of small molecules used in dual inhibition of sEH in combination chemotherapy.

While cisplatin is among the most prominent chemotherapeutic agents, cisplatin-based therapy is highly toxic, underlying the need for improvement in efficacy of cisplatin-therapies [155]. The modulation of the arachidonic acid pathway is considered one of the crucial strategies to avoid toxicity associated with cisplatin therapy [156]. The overexpression of COX-2 in tumor or stromal cells is responsible for tumor angiogenesis [157]. COX-2 inhibitors block production of angiogenic factors, leading to the inhibition of proliferation, migration and vascular tube formation. Nevertheless, coxibs failed in human clinical trials for several cancers [158,159]. Furthermore, sEHIs synergize the analgesic and anti-inflammatory effects associated with coxibs, Refs. [129,130] prevent gastrointestinal erosion, Ref. [131] and alter prostacyclin (PGI2) and thromboxane A2 (TBX2) ratios associated with blood clotting [129]. Thus, the dual COX-2/sEH inhibition strategy can maximize antitumor activity and lower toxic effects of selective COX-2 inhibition, and ultimately may also protect normal tissues from cisplatin toxicity. The chemical structures of some small molecules used in dual inhibition of sEH as part of combination chemotherapy are shown in Figure 8. Combined administration of PTUPB (sEH/COX inhibitor) and cisplatin, increased apoptosis and decreased phosphorylation in the MAPK/ERK and PI3K/AKT/mTOR pathways when compared with controls. Notably, PTUPB treatment did not alter platinum-DNA adduct levels, which is the most critical step in platinum-induced cell death [156].

Pancreatic cancer is one of the most lethal malignant neoplasms. Chronic pancreatitis and mutant Kirsten rat sarcoma viral oncogene homolog KRAS gene (Ki-ras2 Kirsten rat sarcoma viral oncogene homolog) are the most common entities involved in pancreatic carcinogenesis, with more than 90% of human pancreatic carcinomas carrying the KRAS gene mutation [160,161]. Mutations of KRAS lead to constitutive activation of KRAS and persistent stimulation of downstream signaling pathways that initiate carcinogenesis via sustained proliferation, metabolic reprogramming, antiapoptosis, remodeling of the tumor microenvironment, evasion of the immune response, cell migration and metastasis [162]. The mutant RAS-activated RAF1 proto-oncogene serine/threonine kinase (c-RAF)-mitogen-activated protein kinase’s kinase (MEK)-extracellular signal-regulated kinases (ERK) pathway appears crucial for initiating carcinogenesis. Signaling through c-RAF-MEK-ERK, but not b-RAF, is shown to be essential for tumor initiation via mutant Kras, while c-RAF is responsible for transmitting signals from mutant KRAS to MEK-ERK [163]. In the process of long-standing chronic inflammation, aberrant metabolites of arachidonic acid play a crucial role in promoting carcinogenesis. EETs are capable of exerting an effective anti-inflammatory effect by reducing cytokine-induced endothelial cell adhesion molecule (VCAM) and reducing nuclear factor kappa-B kinase and nuclear factor kappa-B kinase inhibitor activity. Therefore, the development of a dual c-RAF/sEH inhibitor would be crucial for suppressing mutant Kras-initiated carcinogenesis [164].

ω-3 epoxy fatty acid metabolites derived from ω-3 polyunsaturated fatty acids (PUFAs) metabolism by cytochrome P450 epoxygenases have been suggested to play a crucial role in inhibiting pancreatic cancer growth. Several studies have suggested the importance of ω-3 epoxide as highly potent metabolites against inflammation and carcinogenesis, particularly via targeting inflammatory signals [165,166,167]. The combined use of ω-3 PUFAs with sEHIs offers a promising strategy for pancreatic cancer treatment and prevention. ω-3 epoxy metabolites prevent pancreatic ductal adenocarcinoma (PDAC) growth via inhibition of the RAS/RAF/ MEK/ERK pathway [168]. ω-3 polyunsaturated fatty acids (PUFAs) are considered immunonutrients and are commonly used in the nutritional therapy of cancer patients. Interestingly, the 2017 European Society for Clinical Nutrition and Metabolism (ESPEN) guidelines for cancer patients discuss the use of omega-3 PUFAs for cancer-cachexia treatment, leaving aside other cancer-related complications that could potentially be managed by omega-3 PUFA supplementation [169].

The use of Bleomycin (BLM) in cancer chemotherapy is constrained by its pulmonary toxicity. A recent study showed that AUDA can reduce pulmonary toxicity in the BLM-induced lung fibrosis in mice model [170]. t-AUCB suppresses human glioblastoma cell growth by activating NF-κβ-p65 [171]. The combination of selective COX-2 inhibitors such as celecoxib and a potent sEHI, namely t-AUCB, synergistically suppresses primary tumor growth and metastasis [127,172]. An orally bioavailable COX-2 and sEH dual inhibitor, PTUPB, displayed excellent potency in reducing inflammatory pain and tumor growth in comparison to the combination of celecoxib and t-AUCB [127,132]. PTUPB has been reported to potentiate cisplatin-based treatment without increasing toxicity in vivo, thereby emphasizing the scope of sEHIs in combination chemotherapy [156]. Another report revealed that the sEHIs, TPPU and t-TUCB, nullify a high-fat diet (HFD)-induced colonic inflammation in mice model. This suggests the importance of sEH in obesity-induced colonic inflammation, which is a major risk factor for the progression and development of colorectal cancer [173]. A dual inhibitor of sEH and RAF1 proto-oncogene serine/threonine kinase (c-RAF), t-CUPM, significantly inhibited chronic pancreatitis and reduced pancreatic intraepithelial neoplasms (PanIN). This approach possibly signals a revolutionary development in preventing and treating pancreatic cancer [164,174]. The VEGF receptor and Raf inhibitor sorafenib showed excellent inhibitory properties on hsEH. Sorafenib treatment was found to reverse lipopolysaccharide-induced hypotension based on the anti-inflammatory effect resulting from soluble epoxide hydrolase inhibition [40]. Activity of sorafenib is thought to stabilize endogenous epoxygenated fatty acids (EpFAs) including antihypertensive, anti-inflammatory, and analgesic EETs [129]. These results suggest the possible scope of sorafenib to control highly angiogenic malignancies including renal cancer.

## 8. Conclusions

The ARA pathway and EETs are deeply interlinked with a variety of physiological outcomes ranging from vasodilation, angiogenesis and inflammation. sEH is a crucial component of the ARA pathway, and sEH inhibition forms a classical strategy for blocking the infiltration of inflammatory mediators. Due to the interconnections between sEH activity, angiogenesis and inflammation-induced carcinogenesis, multitarget therapies involving sEHIs hold promise for treatment of inflammation and a variety of cancers. While urea, carbamate and amide derivatives have been widely reported as sEHIs, these scaffolds have also been successfully leveraged for developing dual inhibitors towards sEH and suitable ARA pathway targets such as FLAP, LTA4 hydrolase and 5-LO. Further, the combination of sEHIs and NSAIDs is capable of synergistically suppressing primary tumor growth and metastasis with reduced toxicity. Multikinase inhibitors in combination with sEHIs offer a promising treatment strategy for hepatocellular and renal cell carcinoma. The use of polyunsaturated fatty acids in combination with sEH inhibition, as well as the dual inhibition of sEH and c-RAF, are potent approaches for preventing pancreatic cancer. Thus, small molecule sEHIs can be effectively leveraged as dual inhibitors and as components of combination chemotherapy. The widespread tissue and cytosolic distribution of sEH emphasizes the need for sEHIs possessing superior solubility and metabolic stability that can facilitate sEH inhibition-based multi-target therapies to achieve better results.

## Abbreviations

sEH	soluble epoxy hydrolase
EET	epoxyeicosatrienoic acid
ARA	arachidonic acid
COX	cyclooxygenase
LOX	lipoxygenase
5-LO	5-lipoxygenase
CYP	cytochrome P450
HETEs	hydroxyeicosatetraenoic acids
LTA4	leukotriene A4
hsEH	human soluble epoxy hydrolase
DHETs	dihydroxyeicosatrienoic acid
Sphk1	Sphingosine kinase 1
FLAP	5-lipoxygenase-activating protein
KRAS	Ki-ras2 Kirsten rat sarcoma viral oncogene homolog
sEHIs	soluble epoxy hydrolase inhibitors
DCU	1,3-Dicyclohexylurea
CDU	1-cyclohexyl-3-dodecyl-urea
AUDA	12-(3-adamantan-1-yl-ureido) dodecanoic acid
AEPU	1-adamantanyl-3-(5-(2-(2-ethoxyethoxy)ethoxy)pentyl)urea
APAU	1-(1-Acetypiperidin-4-yl)-3-adamantanylurea
TPPU	1-(1-propanoylpiperidin-4-yl)-3-[4-(trifluoromethoxy)phenyl]urea
PTUPB	4-(5-phenyl-3-(3-[3-(4-trifluoromethyl-phenyl)-ureido]-propyl)S-pyrazol-1-yl) benzenesulfonamide
t-AUCB	trans-4-[4-(3-Adamantan-1-yl-ureido)-cyclohexyloxy]-benzoic acid
t-TUCB	trans-4-[4-[3-(4-Trifluoromethoxy-phenyl)-ureido]-cyclohexyloxy]-benzoic acid
CPTU	1-(1-(cyclopropanecarbonyl)piperidin-4-yl)-3-(4 (trifluoromethoxy)phenyl)urea
TPAU	(1-trifluoromethoxyphenyl-3-(1-acetylpiperidin-4-yl)urea
ACU	1-adamantyl-3-cyclohexylurea
AMAU	N-((1-acetylpiperidin-4-yl)methyl)-N’-(adamant-1-yl)urea
CUPM	5-[4-[3-(4-chloro-3-trifluoromethyl-phenyl)-ureido]-cyclohexyloxy]-pyridine-2-carboxylic acid methylamide
